# Olfactory Decline in Older Adults with Mild Cognitive Impairment with and without Comorbidities

**DOI:** 10.3390/diagnostics11122228

**Published:** 2021-11-29

**Authors:** Katerina Touliou, Nicos Maglaveras, Evangelos Bekiaris

**Affiliations:** 1 Center for Research and Technology Hellas, Hellenic Institute of Transportation, 57001 Thessaloniki, Greece; touliouk@certh.gr (K.T.); abek@certh.gr (E.B.); 2 Laboratory of Computing, Medical Informatics and Biomedical Imaging Technologies, Medical School, Aristotle University of Thessaloniki, 57001 Thessaloniki, Greece

**Keywords:** mild cognitive impairment (MCI), olfactory deterioration, smell decline, comorbidities, digital diagnostics

## Abstract

Over the past two decades, several studies have measured olfactory performance in Mild Cognitive Impairment (MCI). Deficits are observed in multiple olfactory domains, including odour detection threshold, identification, discrimination, and memory. In this study, the psychophysiological Sniffin’ Sticks smell screening test was administered to examine olfactory functioning in 145 older adults with MCI, a group with MCI and chronic comorbid conditions, and a healthy age-matched comparison group. We hypothesised that olfactory performance will deteriorate in the two MCI groups compared to the control group, even after assessing the known contributions of age and gender. The higher olfactory deterioration in the group with the MCI and the comorbidities in the first year disappeared in the second. This could mean that early consideration of the potential effect of other comorbidities that might affect olfaction should be taken and addressed, as they could easily mask the effect of cognitive decline and/or contribute to it. This study also found higher deterioration in smell identification in participants with MCI, as has been found repeatedly in similar research. Olfactory identification seems to be a more robust marker for discriminating people with MCI and without, and even discriminating between those with MCI and having other health problems.

## 1. Introduction

Earlier identification and diagnosis of individuals likely to develop Alzheimer’s disease (AD) is critical for potential intervention and treatment early in the course of the disease. Hence, there has been intense focus on individuals at risk for developing dementia, in particular those with mild cognitive impairment (MCI). To this effect, recent studies of neuropsychological function in MCI aimed at early detection and prevention strategies.

MCI is often regarded as an intermediate step between normal ageing and Alzheimer’s disease (AD), and the prediction of transition from one step to the next has been the objective of several studies in the last decade, with focus being placed lately on olfactory deterioration as a potential prediction marker for moving from MCI to AD [1,2,3,4].

Memory deterioration is related to decline in smell sensation [5]. It is important, though, to keep in mind that olfaction is a sensory modality that deteriorates with age [6], more often in male than female individuals [7] and often with people being unaware of this decline [8,9]. A recent literature review showed that olfactory dysfunctions are a powerful early marker of forthcoming neuropsychiatric, neurodegenerative, and communication disorders [10]. Olfactory decline can be found in 85% of individuals with early Alzheimer’s disease (AD), which makes it an attractive biomarker for the early identification of neurodegenerative diseases such as AD before any pathological implications become evident [11].

Previous research has shown that individuals with cognitive impairment score lower in olfactory functions when compared to controls [8,12]. Other studies have shown conflicting results, especially when the olfactory ability is subjectively measured (i.e., self-reported) [13]. Older people with cognitive impairment tend to over-estimate their olfactory abilities and this over-estimation increases with age and cognitive deterioration [5].

Neuropsychological and cognitive screening batteries remain strong diagnostic tools in MCI and AD. Accurate and cost-effective methods are additionally required to quickly and cheaply predict the onset of AD and the transition from MCI to AD when it happens.

Changes in the olfactory system are often implicated in the pathogenesis of Alzheimer’s Disease (AD). Over the past two decades, several studies have measured olfactory performance in MCI. Deficits are observed in multiple olfactory domains, including odour detection threshold, identification, discrimination, and memory.

This is a study in the Greek population in a period of two years (Phase I and Phase II examinations). We examined olfactory functioning in adults with MCI and a group with MCI and chronic comorbid conditions, and compared them with a healthy age-matched comparison group. We hypothesise that olfactory performance will be lower in MCI as compared with controls, even after assessing the known contributions of age and gender.

## 2. Materials and Methods

The sample was drawn from community dwelling adults in the city of Thessaloniki and the surrounding regional Thessaloniki Prefecture, Greece, who were recruited from primary and tertiary care clinics. Recruitment targeted individuals with mild memory concerns, as well the general community of older citizens, with the aim of obtaining an MCI-enriched sample as well as older people with MCI. We excluded participants who reported a prior diagnosis of dementia (*n* = 8). Of 153 respondents, 145 participated and met the criteria for this study. The age-range was 56–84 (mean = 67.35, SD = 6.29, 29% female). Overall, 42 female individuals participated in the study (Control: 11, MCI: 17, MCI and comorbid: 14). MCI diagnosis was performed according to Petersen’s criteria [14]. The study was approved by the Bioethics Committee of CERTH (ETH.COM-17; 15 June 2016). 

The Sniffin’ Sticks (Burghardt^®^, Wedel, Germany) were used for three separate olfactory tests: (a) threshold test, (b) discriminating test, and (c) identification test, and they allow semi-objective assessment of the patient’s olfactory performance by means of these three subtests. The sticks are used to investigate olfactory performance. The Extended Test (also called “TDI-Test“—Threshold, Discrimination, Identification) consists of sub-tests with which the ability to identify and discriminate smells is tested and a subtest tests the smell threshold. The complete test takes 30–40 min. The results of all subtests are added up to obtain the so called “TDI-score”. The 3 parts of the Extended Test were applied in the following order: (1) Threshold Test, (2) Discrimination Test, and (3) Identification Test. A break of about three minutes was made between the three tests [15].

**Figure 1:**
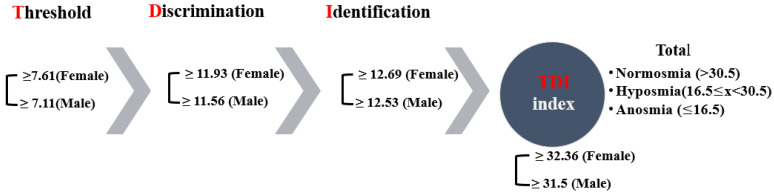
The olfactory psychophysiological testing process and respective norms.

The examiner always wore odourless gloves (e.g., made from cotton) and changed them for each participant. When testing, the cap of only one pen was removed. For testing, the opened odour pen is held 2 cm centered in front of both nostrils and the patient is asked to smell, e.g., using the word “attention”. Each pen should not be presented for more than 3–4 s. The interval between the different presentations was around 30 s. The odour pen did not touch the patient’s skin during the smell presentation. The participants were instructed not to have anything but water for at least 15 min before the test. They were asked to refrain from consuming chewing gum, sweets, or cigarettes. The testing sessions were held in a quiet and well-ventilated room. During the briefing session, participants were asked about their occupational background, gender, age, medical history, drug use, and smoking habits. Participants received no unintentional hints about the correctness of their decisions [15]. The result of this test is expressed as the sum of the results of the 3 subtests, the so called TDI score (threshold, discrimination, identification) (Figure 1). Here, a score of more than 30 rates as normal, a score of 30 or less indicates hyposmia, and a score of 15 and below points to functional anosmia in the form of a complete loss of the sense of smell or an extremely weakened smell ability (Figure 1). The Greek-validated version [16] of the test was administered by replacing certain words to accommodate for cultural appropriateness. The norms for female and male participants, which are over >55 years of age, are shown per olfactory test in Figure 1, as well as the overall thresholds for the TDI index.

### Procedure

The patients were familiarised with the smell of the target pen. For the actual test the patients are blindfolded. Three pens are presented to the participant and they have to find the pen that smells different than the two other pens. The presentation order of the pen containing the odorant has to be alternated by the examiner within each triplet.

(1) Threshold: A certain odorant concentration is only identified correctly if the pen containing the odorant is recognised twice in a row, that is, if the pen containing the odorant is identified again in a second presentation of the same triplet (although a triplet is only presented a second time if the patient has identified the odorant correctly during the first presentation). After that, the next higher dilution step is presented. If this is also identified correctly twice, again the next higher dilution step is presented and so on, until the participant makes a wrong decision. This procedure goes on until certain steps are satisfied.
(2) Discrimination Test: Pens are presented in the form of triplets and are presented to the participants with only one pen holding a different smell, and the participant has always to make a choice. The number of correct responses define the score of this test.
(3) Identification Test: The participant gets a card with four terms each (multiple-choice card). It is the patient’s task to pick the term which describes the presented odorant best. The terms are provided in a separate protocol sheet available to the researcher [15].

## 3. Results

Tests were performed two times; once in phase I and then after a year for phase II, to investigate any changes in ability to smell between the two phases. Data were collected from all groups in both phases and comparisons were made between the groups within a phase, between phases, and within the same group across phases.

Three levels of change in olfactory ability were measured: (a) threshold, (b) discrimination, and (c) identification. Multivariate Analysis of Variance (MANOVA) was applied to investigate differences between the three groups and corrected with Bonferroni post-hoc tests. The Standard Error of the Mean (SEM) is used instead of Standard Deviation (SD) to present the precision of the sample throughout this paper unless stated otherwise. As parametric tests have been applied after the relevant assumptions (i.e., normality, sphericity, randomness, multivariate normality) have been tested, then SEM seems to be a more appropriate descriptive statistic to be used to depict dispersion, and is a better depiction of the precision of the mean measurement and mean differences are of interest for the comparisons at hand.

### 3.1. Threshold

**Figure 2:**
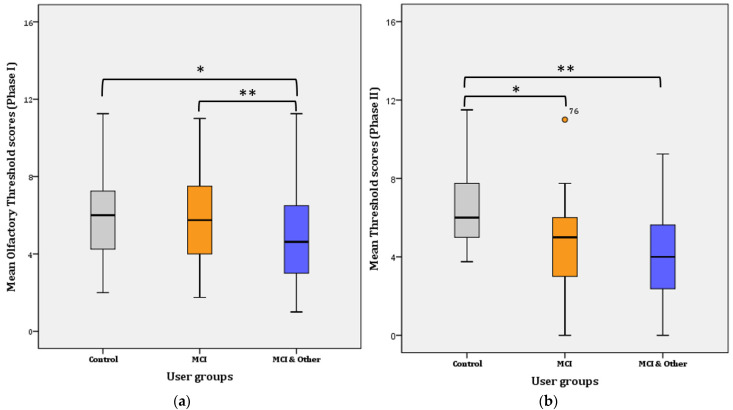
Mean Threshold scores per user group: (**a**) for Phase I; * for Control–MCI, *p* = 0.014, ** for Control–MCI & Other, *p* < 0.001. (**b**) for Phase II; * for Control–MCI, *p* < 0.001, ** for Control–MCI & Other, *p* < 0.001.

A two-way repeated measures ANOVA was performed to evaluate the difference in olfactory threshold levels among the three user groups and across time (i.e., one year passed from Phase 1 to Phase 2). The assumptions related to the application of normal distribution statistical tests were not violated. Overall, a statistically significant decrease of 0.469 in mean threshold scores was found a year after the first test, from 5.468 (SE = 0.184) to 4.999 (SE = 0.194), (F(1, 144) = 4.868, *p* = 0.029, η^2^ = 0.033, Observed power = 0.592). Likewise, an overall significant difference was found in Phase I (F(2, 145) = 4.881, *p* = 0.009, η^2^ = 0.63, Observed power = 0.797) and Phase II (F(2, 144) = 16.908, *p* < 0.001, η^2^ = 0.192, Observed power = 1), respectively. Therefore, significant mean differences were revealed among the three groups in Phase I and among the three groups in Phase II. Further pairwise comparisons were conducted and the results per Phase I are presented below in Figure 2.

Phase I: Further Bonferroni adjustment revealed that the significance lies in the mean difference (1.283 ± 0.447) between the control and the MCI & Other group (*p* = 0.014; noted with * in Figure 2a) and the MCI and the MCI & Other group (1.956 ± 0.376; *p* < 0.001; noted with ** in Figure 2a). The difference between the control and the MCI (0.22 ± 0.458) groups was not statistically significant (*p* > 0.017; adjusted for pairwise comparisons) ((a) for Phase I and (b) for Phase II in Figure 2).

Phase II: Further Bonferroni adjustment revealed that the significance lies in the mean difference (2.022 ± 0.482) between the control and the MCI group (*p* < 0.001; noted with * in Figure 2b) and the control and the MCI & Other group (2.686 ± 0.477; *p* < 0.001; noted with ** in Figure 2b). The difference between the MCI and the MCI & Other groups (0.664 ± 0.467) disappeared and it was not statistically significant (*p* > 0.017; adjusted for pairwise comparisons).

Overall, mean threshold scores increased (by 0.618) in the control group from Phase I (5.95 ± 0.315; CI: 5.315–6.585) to Phase II (6.568 ± 0.303; CI: 5.957–7.179). The difference is not statistically significant (F(1, 44) = 3.396, *p* = 0.072); however, a trend of increase is evident. On the contrary, the mean threshold scores significantly decreased in the MCI (mean decrease = 1.184; F(1, 48) = 17.427, *p* < 0.001), but not in the MCI & Other group (mean decrease = 0.843; F(1, 50) = 17.427, *p* < 0.087). The latter tends towards significance, however, because increase was found in the ‘younger’ age group (i.e., 51–60 years), statistically significant decrease was not obtained.

Likewise, most participants are below the norm in Phase II, with the significance becoming significant (χ^2^ (2) = 12.632, *p* = 0.002) (Table 1). However, 13% more participants were above the norm in the control group in Phase II. Statistically significant different proportions with Bonferroni adjusted α level at 0.0083 were found in the control and the MCI & Other groups (*p* = 0.0009 and *p* = 0.0061, respectively) (Table 1).

### 3.2. Discrimination

**Figure 3:**
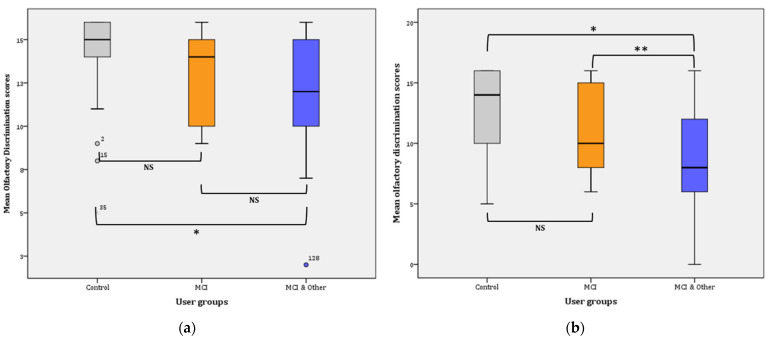
Mean olfactory discrimination scores: (**a**) for Phase I; * *p* < 0.001. (**b**) for Phase II; * *p* < 0.001, ** *p* = 0.016. NS: non-significant.

Overall, discrimination ability significantly declined from Phase I (13.18 ± 0.23) to Phase II (10.86 ± 0.33), (F(1, 147) = 56.559, *p* < 0.001, η^2^ = 0.278, observed power = 1). Phase I: the statistical significance pertains in the comparisons between the three groups in Phase I as is evidenced by the results of the General Linear Mode (GLM): (F(2, 145) = 7.740, *p* = 0.001, η^2^ = 0.096, observed power = 0.946). Further comparisons (Bonferroni adjusted α level = 0.017) revealed significant difference between the control and the MCI & Other group ($\bar{x}$ = 2.13, *p* < 0.001; noted with * in Figure 3a) and no significant difference between the control group and the MCI group ($\bar{x}$ = 1.42, *p* > 0.017; NS). Similarly, the difference between the MCI and the MCI & Other group was not statistically significant ($\bar{x}$ = 0.704, *p* > 0.17; NS) (Figure 3a).

Phase II: the statistical significance pertains in the comparisons between the three groups in Phase I as is evidenced by the results of the GLM: (F(2, 145) = 13.947, *p* < 0.001, η^2^ = 0.161, observed power = 0.998). Further comparisons (Bonferroni adjusted α level = 0.017) revealed a significant difference between the control and the MCI & Other group $\bar{x}$ = 3.889, *p* < 0.001; noted with * in Figure 3b) and no significant difference between the control group and the MCI group ($\bar{x}$ = 1.848, *p* > 0.017; NS). Similarly, the difference between the MCI and the MCI & Other group was marginally statistically significant ($\bar{x}$ = 2.041, *p* = 0.016; noted with ** in Figure 3b).

Pairwise comparisons among the user groups over time showed that significant differences were found between the control group and the experimental groups, i.e., marginal for the MCI ($\bar{x}$ = 1,635, *p* = 0.009 **) and greater when compared to the MCI & Other group ($\bar{x}$ = 3.007, *p* < 0.001 *). However, the difference between the two experimental groups was not statistically significant ($\bar{x}$ = 1.372, *p* = 0.028; NS). The Bonferroni adjusted α was set at 0.0083.

### 3.3. Identification

Overall, identification ability significantly declined from Phase I (9.93 ± 0.17) to Phase II (8.99 ± 0.19), (F(1, 129) = 56.559, *p* < 0.001, η^2^ = 0.111 observed power = 0.986). 

**Figure 4:**
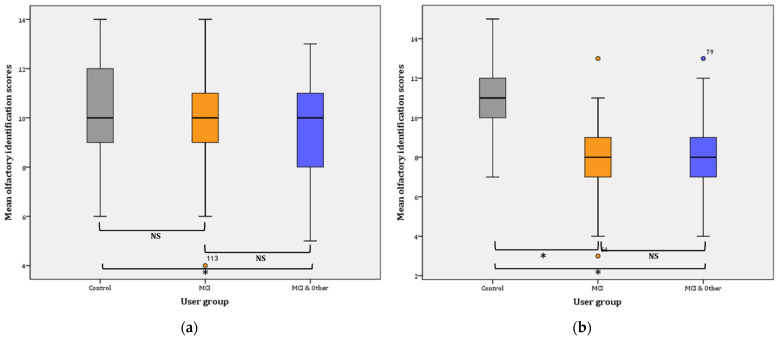
Mean olfactory identification scores: (**a**) for Phase I; for Control–MCI & Other, * *p* < 0.001. (**b**) for Phase II; for Control–MCI and Control–MCI & Other, * *p* < 0.001, NS: non-significant.

Phase I: the statistical significance pertains in the comparisons between the three groups in Phase I, as evidenced by the results of the GLM: (F(2, 137) = 7.818, *p* = 0.001, η^2^ = 0.102, observed power = 0.948). Further comparisons (Bonferroni adjusted α level = 0.017) revealed a significant difference between the control and the MCI & Other group ($\bar{x}$ = 1.33, *p* < 0.001; noted with * in Figure 4a), and no significant difference between the control group and the MCI group ($\bar{x}$ = 0.748, *p* > 0.05; NS). Similarly, the difference between the MCI and the MCI & Other group was not statistically significant ($\bar{x}\;$= 0.585, *p* > 0.05; NS) (Figure 4a).

Phase II: the statistical significance pertains in the comparisons between the three groups in Phase I as evidenced by the results of the GLM: (F(2, 145) = 38.750, *p* < 0.001, η^2^ = 0.348, observed power = 1). Further comparisons (Bonferroni adjusted α level = 0.017) revealed a significant difference between the control and the MCI & Other group ($\bar{x}$ = 2.937, *p* < 0.001; noted with * in Figure 4b), and significant difference between the control group and the MCI group ($\bar{x}$ = 2.978, *p* < 0.001; noted again with * in Figure 4b). On the contrary, the difference between the MCI and the MCI & Other group was not statistically significant ($\bar{x}$ = 0.041, *p* ≥ 0.05; NS); Figure 4b.

Overall, mean identification scores slightly decreased (by 0.359) in the control group from Phase I (10.33 ± 0.329; CI: 9.667–11) to Phase II (9.974 ± 0.395; CI: 9.175–10.774). The difference is not statistically significant (F(1, 38)= 0.749, *p* = 0.392). On the contrary, the mean identification scores significantly decreased in the MCI (mean decrease = 1.563; F(1, 47) = 16.401, *p* < 0.001). Likewise, a statistically significant decrease was observed for the MCI & Other group (mean decrease = 0.887; F(1, 52) = 5.025, *p* < 0.029).

### 3.4. T(hreshold)D(iscrimination)I(dentification) Index

The TDI index is the sum of threshold, detection, and identification scores. It is the index used to estimate if the individual has normal olfaction (≥30), hyposmia (<30), or anosmia (≤15; slightly higher when adjusted for the Greek sample (Figure 1)). The overall repeated GLM was statistically significant (F(1, 137) = 178.457, *p* < 0.001, η^2^ = 0.723, observed power = 1). Overall, the TDI index significantly decreased (3.34 ± 0.419) from Phase I to Phase II (*p* < 0.001; 95% CI for difference: 2.51–4.17). Mean TDI indexes were both below normal for Phase I (28.54 ± 0.284) and Phase II (25.2 ± 0.318), respectively. The whole sample appears to by hyposmic.

**Figure 5:**
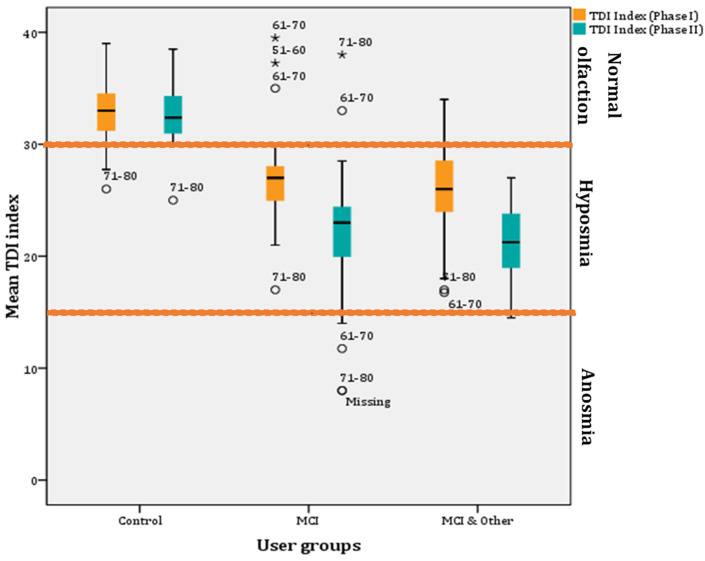
Mean TDI indexes per user group across phases (* for outliers).

Further, investigation of the mean TDI indexes between the groups showed that hyposmia is present only in the experimental groups (i.e., MCI and MCI & other groups). In particular, the pairwise comparisons showed statistically significant differences between the control and the MCI groups (8.33 ± 0.34; *p* < 0.001; 95% CI: 7.02–9.64) and between the control and the MCI & Other group (9.35 ± 0.54; *p* < 0.001; 95% CI: 8.06–10.65), but not between the MCI and MCI & Other group (*p* > 0.05) (Figure 5).

Furthermore, a decrease in mean TDI index scores was found after a year in all groups. However, the decrease is very small (0.415) for the control group and non-significant (*p* > 0.05). However, a statistically significant decrease in mean TDI index (5.05) was found in the MCI group after a year (F(1, 48) = 34.326, *p* < 0.001; η^2^ = 0.417, observed power = 1; 95% CIs of mean difference: 3.314–6.778) and the MCI & Other group (4.571; F(1, 48) = 50.059, *p* < 0.001; η^2^ = 0.511, observed power = 1; 95% CIs of mean difference: 3.272–5.871) (Figure 5).

Phase I: statistically significant differences in the proportions of participants in each user group were found (χ^2^(4) = 92.041, *p* < 0.001). Statistically significant different proportions—with Bonferroni adjusted α level at 0.0067—were found for the pairwise comparisons for the control group for normal (*p* < 0.001) and hyposmia (*p* < 0.001) groups but not for the anosmia group (*p* > 0.05). The results for the MCI (normal with *p* = 0.00035, hyposmia with *p* = 0.00023 and anosmia with *p* > *0*.05) and MCI & Other (normal with *p* < 0.001, hyposmia with *p* = 0.00001 and anosmia with *p* > 0.05) groups were similar (Table 1).

Phase II: Statistically significant differences in the proportions of participants in each user group were found (χ^2^(4) = 115.521, *p* < 0.001). Statistically significant different proportions—with Bonferroni adjusted α level at 0.0067—were found for the pairwise comparisons for the control group for normal (*p* < 0.001) and hyposmia (*p* < 0.001) groups but not for the anosmia group (*p* > 0.05). The results for the MCI (normal with *p* = 0.00035, hyposmia with *p* = 0.00347 and anosmia with *p* > 0.05) and MCI & Other (normal with *p* < 0.001, hyposmia with *p* < 0.001 and anosmia with *p* > 0.05) groups were similar (Table 1)).

**Figure 6:**
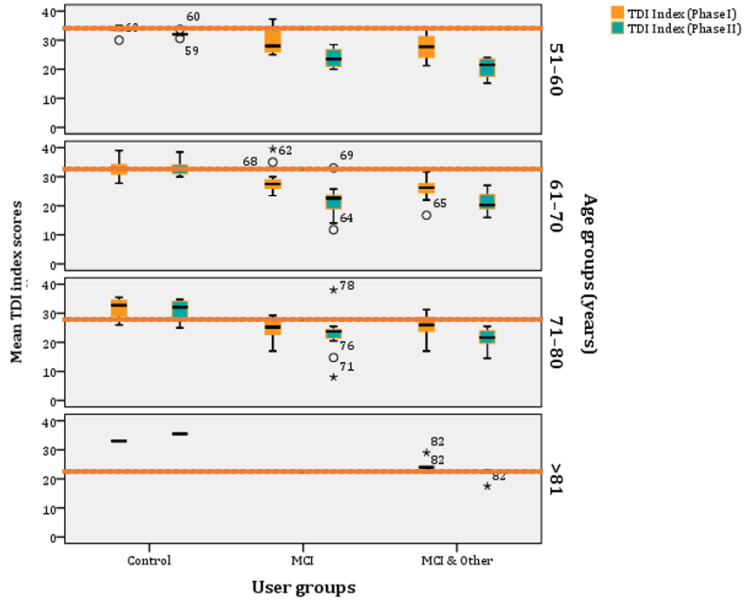
Age–stratified mean TDI index scores per experimental group (* stands for outliers).

If we age-stratify the TDI scores, according to norms as they have been refined by Oleszkiewicz and colleagues [17], they are the following: (a) 51–60 years old: 32.85, (b) 61–70 years old: 31.26, (c) 71–80 years old: 27.93, and (d) >81 years old: 23.30. These are the norms and, below those, people are classified as ‘hyposmic’. Across all age groups, participants are categorised as ‘functional anosmics’ when their mean TDI index is below 16. The MCI and MCI & Other groups’ mean TDI indexes are below the norm across all age-strata, whereas the control group is above the norms for most phases and age clusters (Figure 6).

## 4. Discussion

The current findings are generally consistent with previous research in MCI and AD, where MCI participants showed robust deficits across olfactory domain.

### 4.1. Threshold

The identification of the smell threshold, i.e., the lowest concentration where the participant could identify the presence of the smell, requires important cognitive processes to take place, although it has been found to be affected in AD [3] and usually one year before AD appears [17]. Patients with MCI and AD scored lower on odor discrimination and identification than controls. In our study, this difference was not found; however, it was found only for the comorbid group, which might mean that the MCI group in this study does not show great deterioration in the peripheral part of the olfactory system, or this might not be so detectable yet. Another study [18] did not find any significant deterioration in the MCI when compared to the controls, but found significant deterioration in the identification and discrimination functions, similar to the findings of the Phase I study. It has been advocated that this finding might mean that memory decline is the cause of the difference rather than the pathology. However, after one year, the scenery changed and the MCI significantly deteriorated when compared to the control, and the same holds true for the comorbidity group. After one year, olfactory deterioration was evident and in agreement with relevant research [19], whereas the initial decline, that appeared as a result of the chronic conditions, disappeared. According to this finding, early consideration of the potential effect of other comorbidities that might affect olfaction should be considered and addressed, as they could easily mask the effect of cognitive decline and/or contribute to it.

The sensitivity of smell that is investigated by the threshold olfactory test appears to be affected later in MCI participants, as they did not significantly perform worse than the control group. However, the presence of other chronic conditions significantly affects the ability to detect smell because both the MCI and the control group were significantly better than the third group (i.e., the one with both MCI and chronic conditions).

### 4.2. Discrimination

Several studies have found decline in olfactory discrimination ability in MCI and AD individuals [20,21,22]. On the contrary, another study did not find any differences between MCI patients and the controls [23].

Decline is evident in all groups from Phase I to Phase II; however, in Phase I, there is no significant difference in discrimination scores between the control and MCI, but the chronic conditions appear to be responsible for the discrimination differences, as it was the case for the threshold scores. After one year, both experimental groups show deterioration when compared to the control group. Age appears to be a significant factor in the MCI group.

### 4.3. Identification

Deterioration in olfactory identification has been found repeatedly in research with MCI participants, while results on threshold detection and ability to discriminate odours are inconsistent [24,25], with those with identification problems being at greater risk of progressing to AD than those without [26,27,28]. This study also found higher deterioration in smell identification in participants with MCI.

Albers and colleagues [29] found that, when comparing the sensitivity and specificity of different olfactory tests to more expensive techniques and/or biomarkers, they are similar. Olfactory identification seems to be a more robust marker for discriminating people with MCI and without, and even those between MCI and having other health problems. Previous studies showed that odour identification had good sensitivity and specificity in distinguishing MCI or AD from healthy controls [3,30,31,32,33].

Jung and colleagues [34] performed a meta-analysis and found that olfactory identification tests demonstrated larger effects (d = 0.71, 95% CI: 0.51, 0.91) than tests of other olfactory domains (i.e., odour discrimination and threshold). They also found, in reviewed studies, that male participants showed a greater rate of olfactory deficit than older female participants. Our findings agree with current research and validate this finding of, even when considering comorbidities, which role should be considered systematically, especially in older populations with high prevalence of other chronic conditions. They might act as an accelerator, depending on the condition and its potential effect on the olfactory system or vice versa.

Consideration for different MCI sub-types such as the amnesic have not made it into this study; however, studying the participants in a period of almost two years is important for creating a sound methodological framework. It has been that, for individuals with no cognitive deficits, decline in odour identification can predict that individuals will have MCI (amnestic type) in less than seven years [34,35,36,37,38,39]. An advantage of this study is that the olfactory testing and deterioration is investigated in a longitudinal study; however, larger cohorts with consideration, also, for imaging along with the olfactory, neurological, and cognitive screening would enhance sensitivity and specificity of the diagnosis. It is true that a single biomarker might not suffice for diagnosis of MCI, which is complex and multifactorial in nature. Standardising a battery of several biomarkers would most probably be required for the diagnosis and prediction of MCI and its transition to AD with increased sensitivity and specificity.

## 5. Conclusions

It appears that olfactory psychophysiological tests can act as useful and sensitive screening instruments for MCI and AD individuals before the manifestation of any clinical symptoms that could complement and enhance the sensitivity of existing neuropsychological batteries.

As the interest for simple, fast, and inexpensive biomarkers of AD is avid nowadays, olfactory deterioration opens the way for other sensory impairments to be considered (e.g., auditory dysfunction assessment in AD). Further research is needed to establish the sensitivity and specificity of olfactory tests against other already established measures (e.g., neuropsychological, neurological), without excluding the possibility of their use as initial assessment indicators. To do so, shorter but still robust versions of the olfactory tests could be created to be used during routine physical examinations.

## Figures and Tables

**Table 1:** Percentage (%) of participants per olfactory category in each participant group and across phases.

TDI Category Group	Phase I			Phase II			*p*-Value	
	Normal	Hyposmia	Anosmia	Normal	Hyposmia	Anosmia	I	II
Control	87%	13%	-	95%	5%	-	S *	S
MCI	13%	89%	-	8%	82%	-	S	S
MCI + Co	4%	94%	2%	2%	92%	6%	S	S

* S: Statistically significant comparison at Bonferroni adjusted α level.

## Data Availability

Data sharing not available.

## References

[1] Lehrner J.P., Brücke T., Dal-Bianco P., Gatterer G., Kryspin-Exner I. (1997). Olfactory functions in Parkinson’s disease and Alzheimer’s disease. Chem. Senses.

[2] Murphy C., Schubert C.R., Cruickshanks K.J., Klein B.E., Klein R., Nondahl D.M. (2002). Prevalence of olfactory impairment in older adults. JAMA.

[3] Djordjevic J., Jones-Gotman M., De Sousa K., Chertkow H. (2008). Olfaction in patients with mild cognitive impairment and Alzheimer’s disease. Neurobiol. Aging.

[4] Devanand D.P., Michaels-Marston K.S., Liu X., Pelton G.H., Padilla M., Marder K., Bell K., Stern Y., Mayeux R. (2000). Olfactory deficits in patients with mild cognitive impairment predict Alzheimer’s disease at follow-up. Am. J. Psychiatry.

[5] Wehling E.I., Lundervold A.J., Nordin S., Wollschlaeger D. (2016). Longitudinal changes in familiarity, free and cued odor identification, and edibility judgments for odors in aging individuals. Chem. Senses.

[6] Doty R.L., Kamath V. (2014). The influences of age on olfaction: A review. Front. Psychol..

[7] Kern D.W., Wroblewski K.E., Schumm L.P., Pinto J.M., Chen R.C., McClintock M.K. (2014). Olfactory function in wave 2 of the national social life, health, and aging project. J. Gerontol. Ser. B Psychol. Sci. Soc. Sci..

[8] Bahar-Fuchs A., Moss S., Rowe C., Savage G. (2011). Awareness of olfactory deficits in healthy aging, amnestic mild cognitive impairment, and Alzheimer’s disease. Int. Psychogeriatr..

[9] White T.L., Sadikot A.F., Djordjevic J. (2016). Metacognitive knowledge of olfactory dysfunction in Parkinson’s disease. Brain Cogn..

[10] Bhatia-Dey N., Heinbockel T. (2021). The Olfactory System as Marker of Neurodegeneration in Aging, Neurological and Neuropsychiatric Disorders. Int. J. Environ. Res. Public Health.

[11] Dan X., Wechter N., Gray S., Mohanty J.G., Croteau D.L., Bohr V.A. (2021). Olfactory dysfunction in aging and neurodegenerative diseases. Aging Res. Rev..

[12] Woodward M.R., Amrutkar C.V., Shah H.C., Benedict R.H., Rajakrishnan S., Doody R.S., Yan L., Szigeti K. (2016). Validation of olfactory deficit as a biomarker of Alzheimer disease. Neurol. Clin. Pr..

[13] Devanand D.P., Lee S., Manly J., Andrews H., Schupf N., Doty R.L., Stern Y., Zahodne L.B., Louis E.D., Mayeux R. (2015). Olfactory deficits predict cognitive decline and Alzheimer dementia in an urban community. Neurology.

[14] Petersen R.C. (2004). Mild cognitive impairment as a diagnostic entity. Journal of Internal Medicine.

[15] Rumeau C., Nguyen D.T., Jankowski R. (2016). How to assess olfactory perfor-mance with the Sniffin’Sticks test^®^. Eur. Ann. Otorhinolaryngol. Head Neck Dis..

[16] Konstantinidis I., Printza A., Genetzaki S., Mamali K., Kekes G., Constantinidis J. (2008). Cultural adaptation of an olfactory identification test: The Greek version of Sniffin’Sticks. Rhinology.

[17] Oleszkiewicz A., Schriever V.A., Croy I., Hähner A., Hummel T. (2019). Updated Sniffin’ Sticks normative data based on an extended sample of 9139 subjects. Eur. Arch. Oto-Rhino-Laryngol..

[18] Doorduijn A.S., de van der Schueren M.A.E., van de Rest O., de Leeuw F.A., Fieldhouse J.L.P., Kester M.I., Teunissen C.E., Scheltens P., van der Flier W.M., Visser M. (2020). Olfactory and gustatory functioning and food preferences of patients with Alzheimer’s disease and mild cognitive impairment compared to controls: The NUDAD project. J. Neurol..

[19] Roalf D.R., Moberg M.J., Turetsky B.I., Brennan L., Kabadi S., Wolk D.A., Moberg P.J. (2017). A quantitative meta-analysis of olfactory dysfunction in mild cognitive impairment. J. Neurol. Neurosurg. Psychiatry.

[20] Doty R.L., Doty R.L. (2003). Odor Perception in Neurodegenerative Diseases. Handbook of Olfaction and Gustation.

[21] Kotecha A.M., Correa A.D.C., Fisher K.M., Rushworth J.V. (2018). Olfactory dysfunction as a global biomarker for sniffing out Alzheimer’s disease: A meta-analysis. Biosensors.

[22] Niccoli-Waller C.A., Harvey J., Nordin S., Murphy C. (1999). Remote odor memory in Alzheimer’s disease: Deficits as measured by familiarity. J. Adult Dev..

[23] Kim H.R., Kim S.M., Seong W., Min H.J., Kim K.S., Ga H., Han D.H. (2020). Cut-Off Scores of an Olfactory Function Test for Mild Cognitive Impairment and Dementia. Psychiatry Investig..

[24] Rahayel S., Frasnelli J., Joubert S. (2012). The effect of Alzheimer’s disease and Parkinson’s disease on olfaction: A meta-analysis. Behav. Brain Res..

[25] Mesholam R.I., Moberg P.J., Mahr R.N., Doty R.L. (1998). Olfaction in neurodegenerative disease. Arch. Neurol..

[26] Josefsson M., Larsson M., Nordin S., Adolfsson R., Olofsson J. (2017). APOE-e4 effects on longitudinal decline in olfactory and non-olfactory cognitive abilities in middle-aged and old adults. Sci. Rep..

[27] Devanand D., Tabert M.H., Cuasay K., Manly J.J., Schupf N., Brickman A.M., Andrews H., Brown T.R., DeCarli C., Mayeux R. (2010). Olfactory identification deficits and MCI in a multi-ethnic elderly community sample. Neurobiol. Aging.

[28] Wilson Yaffe K., Freimer D., Chen H. (2017). Olfaction and risk of dementia in a biracial cohort of older adults. Neurology.

[29] Albers M.W., Gilmore G.C., Kaye J., Murphy C., Wingfield A., Bennett D.A., Boxer A.L., Buchman A.S., Cruickshanks K.J., Devanand D.P. (2015). At the interface of sensory and motor dysfunctions and Alzheimer’s disease. Alzheimers Dement..

[30] Chan A., Tam J., Murphy C., Chiu H., Lam L. (2002). Utility of olfactory identification test for diagnosing Chinese patients with Alzheimer‘s disease. J. Clin. Exp. Neuropsychol..

[31] Eibenstein A., Fioretti A., Simaskou M.N., Sucapane P., Mearelli S., Mina C., Amabile G., Fusetti M. (2005). Olfactory screening test in mild cognitive impairment. Neurol. Sci..

[32] Kjelvik G., Sando S.B., Aasly J., Engedal K.A., White L.R. (2007). Use of the Brief Smell Identification Test for olfactory deficit in a Norwegian population with Alzheimer’s disease. Int. J. Geriatr. Psychiatry.

[33] Suzuki Y., Yamamoto S., Umegaki H., Onishi J., Mogi N., Fujishiro H., Iguchi A. (2004). Smell identification test as an indicator for cognitive impairment in Alzheimer’s disease. Int. J. Geriatr. Psychiatry.

[34] Jung H.J., Shin I.S., Lee J.E. (2019). Olfactory function in mild cognitive impairment and Alzheimer’s disease: A meta-analysis. Laryngoscope.

[35] Graves A.B., Bowen J., Rajaram L., McCormick W., McCurry S., Schellenberg G., Larson E.B. (1999). Impaired olfaction as a marker for cognitive decline: Interaction with apolipoprotein E ε4 status. Neurology.

[36] Schubert C.R., Carmichael L.L., Murphy C., Klein B.E., Klein R., Cruickshanks K.J. (2008). Olfaction and the 5−year incidence of cognitive impairment in an epidemiological study of older adults. J. Am. Geriatr. Soc..

[37] Devanand D.P., Lee S., Manly J., Andrews H., Schupf N., Masurkar A., Stern Y., Mayeux R., Doty R.L. (2015). Olfactory identification deficits and increased mortality in the community. Ann. Neurol..

[38] Roberts R.O., Christianson T.J., Kremers W.K., Mielke M.M., Machulda M.M., Vassilaki M., Alhurani R.E., Geda Y.E., Knopman D.S., Petersen R.C. (2016). Association between olfactory dysfunction and amnestic mild cognitive impairment and Alzheimer disease dementia. JAMA Neurol..

[39] Windon M.J., Kim S.J., Oh E.S., Lin S.Y. (2020). Predictive value of olfactory impairment for cognitive decline among cognitively normal adults. Laryngoscope.

